# Combined *Schizochytrium* and Canola Oil Effectively Replaces Fish Oil in Atlantic Salmon (*Salmo salar*) Under Chronic Thermal Stress

**DOI:** 10.1155/anu/8779572

**Published:** 2026-10-30

**Authors:** Suhyeok Kim, Hyunwoon Lim, Jin-Young Jeon, Kyeong-Jun Lee

**Affiliations:** ^1^ Department of Marine Life Sciences Jeju National University Jeju 63243 Republic of Korea jejunu.ac.kr; ^2^ Daesang Corporation Seoul 03130 Republic of Korea; ^3^ Marine Life Research Institute Jeju National University Jeju 63333 Republic of Korea jejunu.ac.kr

**Keywords:** Atlantic salmon, fish-oil replacement, intestinal microbiota, lipid metabolism, *Schizochytrium* oil, sustainable aquaculture, thermal stress

## Abstract

This study evaluated the replacement of fish oil (FO) with *Schizochytrium* oil (SCZO) and canola oil (CNO) in diets of Atlantic salmon (*Salmo salar*) under chronic high‐temperature conditions. Six diets replaced FO at 0%, 20%, 40%, 60%, 80%, and 100% (S0–S100) with SCZO and CNO at a constant replacement ratio of 27.1:72.9 (w/w), while maintaining comparable dietary EPA + DHA levels. Fish (101.01 ± 0.25 g) were stocked at 20 fish per tank with three replicates per treatment and fed for 6 weeks during which water temperature increased from ~15.5 to 25.0°C. Compared with S0, weight gain (WG) was significantly higher in S20–S60, whereas protein‐efficiency ratio (PER) and survival were higher and feed conversion ratio (FCR) was lower in S20–S100. Plasma glucose, triglyceride, aspartate aminotransferase (AST), alanine aminotransferase (ALT), low‐density lipoprotein (LDL), and cortisol were lower in S20–S100 than in S0, whereas high‐density lipoprotein (HDL) was higher in S60–S100. Antioxidant enzyme activities were higher and hepatic malondialdehyde (MDA) was lower in S20–S100, whereas myeloperoxidase (MPO), lysozyme, and respiratory burst activities were higher in S40–S100. Intestinal villi length was increased and vacuolization was decreased in S20–S100. Genes related to fatty acid synthesis, heat‐shock responses, inflammation, and apoptosis were downregulated in S20–S100 compared with S0, whereas fatty acid catabolism‐related genes were upregulated in S20–S100. Muscle DHA was higher in S20–S100 and crude protein in S40–S100 than in S0. Taxonomic profiling showed lower abundances of *Vibrio* and *Photobacterium* in S20–S100 than in S0 and increasing abundances of *Lactobacillus* and *Bacillus* with FO replacement, accompanied by increased alpha diversity and distinct beta‐diversity clustering. Quadratic regression estimated optimal FO replacement levels of 58.6% for WG and 81.6% for survival. Overall, SCZO with CNO effectively replaced FO and supported growth, physiological responses and survival of *S. salar* during chronic high‐temperature exposure.

## 1. Introduction

Fish oil (FO) has traditionally been used as a primary lipid source in aquafeed because of its high levels of long‐chain polyunsaturated fatty acids (LC‐PUFAs), particularly eicosapentaenoic acid (EPA; C20:5n3) and docosahexaenoic acid (DHA; C22:6n3) [1]. However, the limited availability, increasing costs, and sustainability concerns associated with FO have prompted the exploration of alternatives [2]. The replacement of FO without compromising fish health and growth performance remains a major challenge in aquaculture nutrition. Plant‐derived oils such as soybean oil and canola oil (CNO) have been widely used considering their availability and energy content [3], although they lack *n*‐3 LC‐PUFAs, which are vital for maintaining cell membrane integrity, regulating lipid metabolism, supporting immune function, and enhancing stress resistance in fish [4]. Therefore, nonmarine *n*‐3 LC‐PUFA sources are crucial for reducing reliance on marine resources and promoting sustainable aquaculture.

Microalgal oils have gained substantial attention as alternative aquafeed lipid sources because of their high essential fatty acid content and sustainable production, with no reliance on marine resources [5]. Genera such as *Crypthecodinium*, *Nannochloropsis*, *Schizochytrium* (SCZ), and *Tisochrysis* are rich in *n*‐3 LC‐PUFAs, making them promising FO substitutes [6]. In addition, life‐cycle assessments have highlighted the potential of microalgae‐based ingredients to reduce reliance on finite marine resources and lower biotic resource depletion in aquafeed production [7]. In particular, SCZ oil (SCZO) is a stable, bioavailable source of DHA [8]. However, microalgae‐derived oils such as SCZO are rich in DHA but generally contain low levels of C18 fatty acids such as linoleic acid (LA; C18:2n6) and *α*‐linolenic acid (ALA; C18:3n3) compared with those in plant‐derived oils [9]. In contrast, plant‐derived oils are rich in LA and other omega‐6 fatty acids, serving as effective supplements by providing essential C18 fatty acids [10]. Therefore, combining microalgae‐ and plant‐derived oils can help achieve both nutritional balance and physiological benefits [11].

The Atlantic salmon (*Salmo salar*) is an economically important aquaculture species worldwide. However, rising water temperatures linked to climate change increasingly expose farmed fish to thermal stress, negatively affecting growth, health, and physiology [12]. Thermal stress induces oxidative stress, impairs immune responses, disrupts intestinal integrity, and alters gut microbial communities, ultimately reducing aquaculture productivity [13]. Higher water temperatures complicate FO replacement as alternative lipid sources lack comparable functional properties, such as maintaining membrane integrity and regulating inflammation and oxidative responses, which are required to support stress resilience [14]. Notably, *S. salar* has a high lipid requirement (20%–25% of the diet) and depends on PUFA, making FO a primary dietary lipid source [4]; thus, identifying sustainable alternatives is crucial. In this context, the combination of SCZO and CNO may provide a practical FO replacement strategy as a source of DHA and energy, with potential benefits for metabolic regulation and stress resilience [15, 16]. Dietary lipid composition also influences lipid metabolism, immune responses, and intestinal microbiota [17]. However, limited data exist regarding the combined use of SCZO and CNO as dietary FO replacement in *S. salar* under elevated water temperatures. Previous studies have demonstrated impaired growth, physiological disturbances, and increased mortality in *S. salar* exposed to elevated seawater temperatures above ~20°C [18, 19]. Therefore, in the present study, we used 24–25°C as a severe high‐temperature challenge to evaluate SCZO and CNO as alternative lipid sources under a physiologically demanding rearing environment. Studies investigating graded FO replacement with combined lipid sources are scarce, and optimal replacement levels have not been established. Therefore, the present study aimed to evaluate the feasibility of replacing dietary FO with a combination of SCZO and CNO in *S. salar* under high temperature, providing diets containing graded FO replacement levels (0%–100%) and assessing fish growth, physiological responses, antioxidant capacity, immune function, digestive enzyme activity, gene expression, fatty acid composition, and intestinal microbiota.

## 2. Materials and Methods

### 2.1. Experimental Diets

The SCZO used in the present study was supplied by Daesang Corporation (Seoul, South Korea). SCZ sp. was cultivated by fermentation in a sterilized medium containing food‐grade nutrients. Seed cultures were sequentially expanded and transferred to a stainless‐steel main fermenter, where fermentation was conducted at 29°C under controlled conditions. After fermentation, the culture broth was subjected to enzymatic oil extraction at 50–65°C and a pH of 8–10 to facilitate oil release from the biomass. The extracted suspension was then separated into oil, aqueous, and sludge fractions using a three‐phase high‐speed centrifugation system, and the recovered oil fraction was used as the SCZO source in the experimental diets. Thus, SCZO used in this study was an extracted oil product rather than whole‐cell SCZ biomass. Table 1 presents the fatty acid profiles of the lipid sources and experimental diets. To characterize the oxidative quality of the lipid sources used for diet preparation, peroxide value (PV) and *p*‐anisidine value (*p*‐AV) were determined in triplicate at the time of diet preparation, according to Hrebień‐Filisińska and Bartkowiak [20], and total oxidation value (TOTOX) was calculated as 2 × PV + *p*‐AV (Table S1).

**Table 1:** Fatty acid composition (% of total FAME) of the lipid sources and experimental diets for Atlantic salmon *Salmo salar*.

Fatty acids	Lipid sources			Experimental diets					
	FO^a^	CNO^b^	SCZO^c^	S0	S20	S40	S60	S80	S100
SFA^d^									
C14:0	8.67	0.00	4.47	5.24	4.45	3.66	2.86	2.07	1.28
C16:0	18.2	4.29	16.5	14.57	13.4	12.3	11.2	10.1	8.53
C18:0	4.69	0.00	4.52	3.51	3.14	2.78	2.41	2.04	1.67
C21:0	2.71	0.00	0.00	1.44	1.15	0.86	0.58	0.29	0.00
MUFA^e^									
C16:1	12.2	0.22	0.16	7.51	6.23	4.96	3.68	2.40	1.13
C18:1n9	22.6	63.3	0.52	36.5	39.0	41.5	44.0	46.5	49.1
C20:1	12.6	0.00	0.00	6.26	4.95	3.67	2.35	1.05	0.13
PUFA^f^									
C18:2n6	1.51	20.2	0.25	8.48	9.89	11.3	12.82	14.15	15.6
C18:3n3	0.71	12.0	0.58	5.19	6.06	6.92	7.87	8.67	9.56
C20:4n6	1.01	0.00	0.00	1.10	0.96	0.81	0.67	0.52	0.35
C20:5n3	10.8	0.00	0.45	6.54	5.48	4.39	3.29	2.20	1.12
C22:5n6	0.00	0.00	16.5	0.00	0.48	0.95	1.43	1.90	2.38
C22:6n3	4.30	0.00	56.1	3.71	4.77	5.9	6.88	8.13	9.21
∑SFA	34.3	4.29	25.5	24.8	22.2	19.6	17.0	14.5	11.5
∑MUFA	47.4	63.5	0.68	50.3	50.2	50.1	50.0	50.0	50.4
∑PUFA	18.3	32.2	73.9	25.0	27.6	30.3	33.0	35.6	38.2
∑*n*‐3	15.8	12.0	57.1	15.4	16.3	17.2	18.0	19.0	19.9
∑*n*‐6	2.52	20.2	16.8	9.58	11.3	13.1	14.9	16.6	18.3

^a^Fish oil (cod liver oil); E‐Hwa Oil & Fat Industry Corp., Busan, Korea.

^b^Canola oil; UMAC Corp., Haenam, South Korea.

^c^ *Schizochytrium* oil; Daesang Corp., Seoul, South Korea.

^d^Saturated fatty acids.

^e^Monounsaturated fatty acids.

^f^Polyunsaturated fatty acids.

All experimental diets were formulated to be isonitrogenous (44% crude protein) and isolipidic (24% crude lipid). The control diet (S0) contained fish meal (anchovy and sardine meal, 25%) as the primary protein source, along with soy protein concentrate and wheat gluten. FO (commercial cod liver oil; CLO, 12%) and CNO (8%) served as the main lipid sources. In the experimental diets, FO in the S0 diet was progressively replaced with a combination of SCZO and CNO at levels of 20%, 40%, 60%, 80%, and 100% (S20, S40, S60, S80, and S100 diets, respectively) while maintaining consistent dietary EPA + DHA levels. All dry ingredients were thoroughly mixed with the respective dietary oils and distilled water (14% of the total diet weight) using a commercial feed mixer (NVM‐14‐2P, Daeyoung, Seoul, South Korea). The resulting dough was pelletized using a laboratory‐scale compression pellet mill (SP‐75, Kumkang Engineering, Daegu, South Korea) equipped with a 3‐mm die. Thus, the experimental diets were produced as compressed pellets without an extrusion or expansion process. The pellets were subsequently dried and stored at −20°C until use. Table 2 summarizes the formulation, proximate composition, and EPA and DHA levels of the experimental diets.

**Table 2:** Formulation, proximate composition, and essential fatty acid profile of the experimental diets for Atlantic salmon *Salmo salar*.

Ingredients (%, dry matter)	Experimental diets					
	S0	S20	S40	S60	S80	S100
Fish meal (anchovy)	12.5	12.5	12.5	12.5	12.5	12.5
Fish meal (sardine)	12.5	12.5	12.5	12.5	12.5	12.5
Soy protein concentrate	18.5	18.5	18.5	18.5	18.5	18.5
Wheat gluten	16.0	16.0	16.0	16.0	16.0	16.0
Starch	15.0	15.0	15.0	15.0	15.0	15.0
Fish oil (cod liver)^a^	12.00	9.60	7.20	4.80	2.40	0.00
CNO^b^	8.00	9.75	11.50	13.25	15.00	16.75
SCZO^c^	0.00	0.65	1.30	1.95	2.60	3.25
Monocalcium phosphate	1.0	1.0	1.0	1.0	1.0	1.0
Choline chloride	0.5	0.5	0.5	0.5	0.5	0.5
Lecithin	1.0	1.0	1.0	1.0	1.0	1.0
Vitamin mix^d^	1.5	1.5	1.5	1.5	1.5	1.5
Mineral mix^e^	1.5	1.5	1.5	1.5	1.5	1.5
*Proximate composition* (*%*, *dry matter*)						
Crude protein	44.2	44.1	44.2	44.2	44.3	44.1
Crude lipid	24.0	23.9	23.9	24.0	24.1	24.1
Crude ash	8.52	8.52	8.52	8.52	8.52	8.52
C20:5n3 (EPA)	1.57	1.31	1.05	0.79	0.53	0.27
C22:6n3 (DHA)	0.89	1.14	1.41	1.65	1.96	2.22
EPA + DHA	2.46	2.45	2.46	2.44	2.49	2.49

^a^E‐Hwa Oil and Fat Industry Corp., Busan, Korea.

^b^Canola oil; UMAC Corp., Haenam, South Korea.

^c^ *Schizochytrium* oil; Daesang Corp., Seoul, South Korea.

^d^Vitamin premix (g kg^−1^of mixture): retinol, 3.0; cholecalciferol, 1.0; ascorbic acid, 20.0; tocopherol, 20.0; menadione, 2.0; thiamin, 4.0; riboflavin, 6.0; pyridoxine, 5.0; cobalamin, 6.0; inositol, 54.0; pantothenic acid, 12.0; biotin, 0.2; niacin amide, 40.0; and folic acid, 2.0.

^e^Mineral premix (g kg^−1^of mixture): ferrous sulfate, 10.0; copper sulfate, 1.0; zinc sulfate, 30; manganous sulfate, 2.0; cobalt chloride, 10.; potassium iodide, 1.0; potassium, 6.0; and sodium selenite, 0.01.

### 2.2. Feeding Trial

The feeding trial was conducted at the Kidang Marine Research Institute, Jeju National University, South Korea. Experimental fish were obtained from a commercial farm (Saeyeon Aquafarm, Dangjin, South Korea) and acclimated to the experimental conditions for 4 weeks while being fed the S0 diet. After acclimation, fish with an initial mean body weight of 101.01 ± 0.25 g were distributed randomly among 18 circular tanks (450 L), with 20 fish per tank and three replicate tanks per dietary treatment. The tanks were aerated continuously to maintain sufficient dissolved oxygen (DO) levels. Water quality parameters during the feeding trial were as follows: DO, 8.15–8.88 mg/L; pH, 7.40–7.46; salinity, 30.0–30.5 psu; and ammonia, 0.002–0.003 mg/L. Thermal stress was induced by mixing chilled seawater from a cooling system (DA‐3000B, Daeil, Busan, South Korea) with ambient seawater. The fish were maintained at ~15.5°C during acclimation and until day 4 of the feeding trial. From days 5 to 8, the water temperature gradually increased to reach the higher target of ~25.0°C. The 4‐day temperature transition was applied to avoid an immediate transfer from the acclimation temperature to the target high‐temperature condition while establishing the intended thermal challenge during the early phase of the feeding trial. Subsequently, the fish were continuously reared under chronic high‐temperature conditions (24.0–25.0°C) for the remaining 34 days of the 6‐week feeding trial. The water temperature profile during the experimental period is shown in Figure 1. Fish were hand‐fed to apparent satiation three times daily at 08:30, 13:30, and 18:30 h during the trial. Uneaten feed was collected after each feeding, dried in a forced‐air oven at 60°C for 24 h, and weighed. Feed intake (FI) was calculated on a dry‐matter basis by subtracting the dry weight of the uneaten feed from the dry weight of feed offered. FI was adjusted for mortality by excluding the estimated FI attributable to dead fish based on the recorded time of mortality. Thermal stress–induced mortality began on day 9, and the experiment was terminated at 6 weeks when survival in the S0 group approached 50%, as further progressive mortality under sustained high‐temperature exposure could have reduced the number of fish available for the planned physiological, biochemical, histological, molecular, and microbiome analyses and thereby compromised the intended sampling design.

**Figure 1:**
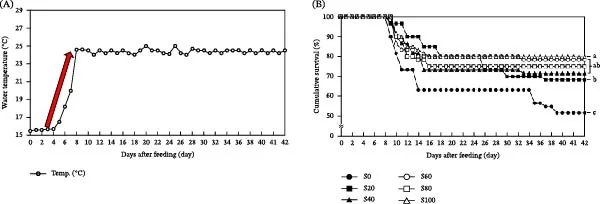
Water temperature profile and cumulative survival of Atlantic salmon *Salmo salar* during the 6 weeks feeding trial. (A) Changes in water temperature during the experimental period. Temperature was increased from ~15.5 to 25.0°C during the initial stage of the trial (arrow) to induce thermal stress. (B) Cumulative survival (%) of fish fed diets containing graded replacement levels of fish oil with *Schizochytrium* oil and canola oil (S0–S100). Values are means of replicate tanks. Different letters indicate significant differences among treatments at the end of the experiment (*p* < 0.05).

### 2.3. Growth Performance, Feed Efficiency, Survival, and Sample Collection

At the end of the feeding trial, all fish were weighed individually to determine growth performance, including final body weight (FBW), weight gain (WG), and specific growth rate (SGR). FI and WG during the trial were used to calculate the feed conversion ratio (FCR) and protein‐efficiency ratio (PER). Survival was monitored throughout the study. After the final weighing, 10 fish per tank (30 fish per treatment) were randomly sampled and anesthetized using 2‐phenoxyethanol (100 ppm). Whole blood samples were collected from the caudal vein using heparinized syringes, transferred into heparinized tubes, and centrifuged at 3000 ×*g* and 4°C for 10 min to extract plasma. All plasma and tissue samples were immediately snap‐frozen in liquid nitrogen and subsequently stored at −80°C until further analyses. Blood samples from all 10 fish per tank were used for hematological, nonspecific immune, and antioxidant analyses. Following blood collection, liver and muscle samples were collected from all 10 fish per tank for hepatic malondialdehyde (MDA) and fatty acid *β*‐oxidation, gene expression, and muscle fatty acid and proximate composition analyses, as appropriate. The anterior intestine and stomach were collected from five fish per tank for digestive enzyme analyses, and the midintestine was collected from five fish per tank for histopathological analysis. For intestinal microbiome analysis, the anterior and midintestinal samples were collected from five fish per tank and pooled within each tank to generate one composite sample.

### 2.4. Hematological Parameters

Plasma biochemical parameters analyzed included glucose, aspartate aminotransferase (AST), alanine aminotransferase (ALT), total cholesterol (CHOL), triglycerides (TGs), high‐density lipoprotein (HDL), low‐density lipoprotein (LDL), and cortisol. Glucose, AST, ALT, CHOL, and TG concentrations were measured using commercial assay kits (Stanbio Labs, Boerne, TX, USA) following the procedures described by Kim et al. [21]. Briefly, 50 µL of plasma was mixed with 1 mL of the respective reagent and incubated at 30°C for 1 min, after which the samples were analyzed using an automatic biochemical analyzer (SLIM, SEAC Inc., Florence, Italy). HDL and LDL levels were measured using commercial enzymatic assay kits (MAK045, Sigma–Aldrich, St. Louis, MO, USA) following the manufacturer’s instructions. Plasma cortisol levels were quantified via ELISA using a commercial assay kit (ab108665, Abcam, Cambridge, UK).

### 2.5. Nonspecific Immune Responses

Nonspecific immune parameters, including immunoglobulin (Ig) levels and myeloperoxidase (MPO), nitro blue tetrazolium (NBT), lysozyme, and antiprotease activities, were analyzed using plasma samples. All assays were performed according to the methods described by Shin et al. [22]. MPO activity was determined using tetramethylbenzidine and H_2_O_2_, with the absorbance being measured at 450 nm. NBT activity was assessed via zymosan‐stimulated respiratory burst activity, followed by reduced formazan solubilization and absorbance measurement at 620 nm. Lysozyme activity was measured using *Micrococcus lysodeikticus* as a substrate, and the decrease in absorbance was monitored at 570 nm. Antiprotease activity was determined using trypsin inhibition and azocasein hydrolysis assays, with the absorbance being measured at 430 nm. Ig levels were estimated after polyethylene glycol precipitation based on the difference between total protein and residual protein concentrations.

### 2.6. Antioxidant Capacity

Antioxidant capacity was evaluated by measuring plasma superoxide dismutase (SOD), glutathione peroxidase (GPx), catalase (CAT), and total antioxidant capacity (TAOC) along with hepatic MDA levels. MDA levels and SOD, GPx, and CAT activities were determined using commercial assay kits (ab118970, Abcam; 19160, Sigma–Aldrich; K762‐100, BioVision, CA, USA; DG‐CAT400, DoGenBio, Seoul, Korea) following the manufacturers’ instructions. Briefly, SOD activity was determined by measuring the inhibition of water‐soluble tetrazolium reduction at 450 nm after incubation at 37°C for 20 min. GPx activity was assessed by monitoring NADPH oxidation at 340 nm, whereas CAT activity was assessed by monitoring the decomposition of hydrogen peroxide, with absorbance measured at 560 nm. Hepatic MDA levels were analyzed using the thiobarbituric acid (TBA) method. Samples were reacted with the TBA reagent at 95°C for 60 min and cooled on ice, and the absorbance was measured at 532 nm. MDA concentrations were calculated using a standard curve and expressed as nmol per sample. TAOC was assessed using the ferric reducing antioxidant power (FRAP) assay following the procedure of Lim et al. [23]. The FRAP reagent was freshly prepared by mixing 0.3 M acetate buffer (pH 3.6), 10 mM 2,4,6‐tripyridyl‐s‐triazine dissolved in 40 mM HCl, and 20 mM FeCl_3_ · 6H_2_O at a ratio of 10:1:1 (v/v/v). Plasma samples were incubated with the FRAP reagent at 37°C for 30 min, and absorbance was measured at 593 nm. A calibration curve was prepared using FeSO_4_ · 7H_2_O standard solutions (0–1200 μmol/L), yielding a linear relationship with a correlation coefficient of 0.9998. TAOC values were calculated from the standard curve and expressed as μmol Fe^2+^ equivalents.

### 2.7. Digestive Enzyme Activity and Fatty Acid *β*‐Oxidation

Digestive enzyme activities were determined in the anterior intestine and stomach tissues of *S. salar*, with trypsin, chymotrypsin, and lipase activities measured in the anterior intestine and pepsin activity in the stomach, following the methods of Natalia et al. [24]. For both tissue types, samples (50 mg) were homogenized in 80 mM Tris–HCl buffer (pH 8.0) and centrifuged at 15,000 ×*g* and 4°C for 15 min. The supernatant was collected, and total protein concentration was determined using a Bio‐Rad protein assay kit (#5000002, Bio‐Rad, CA, USA). Trypsin and chymotrypsin activities were measured using synthetic substrates, with absorbance being recorded at 410 nm. Lipase activity was determined based on the hydrolysis of TG in an olive oil emulsion, followed by titrating the fatty acids released with NaOH. Enzyme activities were expressed relative to the protein level. Pepsin activity was assayed using 2% hemoglobin in 0.06 N HCl as the substrate. Briefly, 100 µL of enzyme extract (in 0.01 N HCl) was incubated with 500 µL of the substrate at 37°C for 10 min. The reaction was terminated by adding 1 mL of 5% trichloroacetic acid (TCA), and the solution was centrifuged at 12,000 ×*g* and 4°C for 5 min. The absorbance was measured at 280 nm, with blanks prepared by adding TCA prior to enzyme addition.

Hepatic fatty acid *β*‐oxidation was measured using a commercial assay kit (#BR00001; Assay Genie, Dublin, Ireland) following the manufacturer’s instructions. Briefly, liver samples (20 mg) were homogenized in lysis buffer and centrifuged at 15,000 ×*g* and 4°C for 5 min, and the protein concentration of the supernatant was adjusted to 1–2 mg/mL using the bicinchoninic acid method. Aliquots of 20 µL were loaded into a 96‐well plate in duplicate, followed by the addition of control and reaction solutions. After incubation at 37°C for 60–120 min, absorbance was measured at 492 nm, and *β*‐oxidation activity was calculated from the absorbance difference between the reaction and control wells (ΔOD) using the manufacturer’s conversion factors.

### 2.8. Histopathological Analysis

Histopathological analysis was performed using midintestinal tissues, measuring villi length, width, and vacuolization (Figure S1). Samples were fixed in 10% neutral formalin for 24 h, dehydrated in a graded ethanol series (70%–100%), cleared in xylene, embedded in paraffin, sectioned (5 µm), and stained with hematoxylin and eosin. The stained sections were observed under a light microscope (DM750, Leica Microsystems, Heerbrugg, Switzerland) equipped with a digital camera (ICC 50 E, Leica Microsystems), and morphological parameters were measured using an image analysis software (Leica Application Suite, v4.13.0).

### 2.9. Gene Expression

Gene expression related to lipid metabolism, stress response, inflammation, and apoptosis was analyzed in the liver of *S. salar*. Target genes were grouped by their physiological functions as follows: fatty acid synthesis, including fatty acid synthase (*FAS*), delta‐5 desaturase (Δ*5D*), delta‐6 desaturase (Δ*6D*), elongation of very long‐chain fatty acids protein 2 (*ELOVL2*), and elongation of very long‐chain fatty acids protein 5 (*ELOVL5*); fatty acid transcription factors, including liver X receptor (*LXR*), sterol regulatory element‐binding protein 1 (*SREBP1*), and sterol regulatory element‐binding protein 2 (*SREBP2*); fatty acid catabolism, including acyl‐CoA oxidase 1 (*ACOX1*), carnitine palmitoyl‐transferase 1 (*CPT1*), and peroxisome proliferator‐activated receptor alpha (*PPARα*), peroxisome proliferator‐activated receptor beta (*PPARβ*), and peroxisome proliferator‐activated receptor gamma (*PPARγ*); heat shock and stress response, including heat‐shock protein 60 (*HSP60*), heat‐shock protein 70 (*HSP70*), heat‐shock protein 90 (*HSP90*), and serpin family H member 1 (*Serpin H1*); inflammation, including tumor necrosis factor alpha (*TNF-α*), interleukin‐1 beta (*IL-1β*), interleukin‐6 (*IL-6*), and mitochondrial ubiquitin ligase activator of NF‐κB (*MULAN*); and apoptosis, including Bcl‐2‐associated X protein (*BAX*), caspase‐3a (*Caspase-3a*), caspase‐7 (*Caspase-7*), caspase‐9 (*Caspase-9*), and cellular tumor antigen p53 (*P53*). The reference genes included *β-actin*, *18S* rRNA, and *EF1α*. Table 3 lists the primer sequences and accession numbers. Total RNA was extracted from liver tissues using the TRIzol reagent (15596026, Thermo Fisher Scientific, MA, USA), and RNA purity was assessed using a NanoDrop 2000 spectrophotometer (Thermo Scientific, Wilmington, DE, USA). First‐strand cDNA was synthesized using the PrimeScript cDNA Synthesis Kit (TaKaRa, Shiga, Japan) and diluted 10‐fold before the analysis. The quantitative polymerase chain reaction protocol began with initial denaturation at 95°C for 5 min, followed by 45 cycles of denaturation at 95°C for 15 s, annealing at 55°C for 25 s, and extension at 72°C for 30 s. Dissociation analysis was then performed at 95°C for 15 s, 60°C for 30 s, and 72°C for 2 min to verify amplification specificity. Relative gene expression levels were calculated using the efficiency‐corrected comparative Ct method [25].

**Table 3:** Primers used for quantitative real‐time PCR.

Function	Gene	Sequence (5′–3′)	Accession number
Fatty acid synthesis	*FAS*	F: GATGCTCAACTCGGTACTG	CK876943.1
		R: CCCCAAAACAAAGACAGCC	
	Δ*5D*	F: CTTCTTCCTCATTGGACCTC	NM_001123542.2
		R: CTGACTTCTTCAGTGACCTG	
	Δ*6D*	F: GGGGATCTGTGGCTAGATGC	NM_001123575.2
		R: CTGACACAAACGTCTGGGGA	
	*ELOVL2*	F: CTCACTCAAGGGTGTCTTAC	NM_001136553.1
		R: GTGGCCATATAGGAGAACTG	
	*ELOVL5*	F: CTATGAGATGGTGTCTGCTG	NM_001136552.1
		R: GAGCCATTCTGGTGATACTC	

Fatty acid transcription factor	*LXR*	F: GCTATCTGAAATCTGGGACG	FJ470290.1
		R: CGATGATGTGGATCTGAGAG	
	*SREBP1*	F: TCCTCTCCCTCAATCCCCTG	HM561860.1
		R: GTTGTCTAGGCCCAGCATGT	
	*SREBP2*	F: CTGGATACACCACGGCAGAG	DY733476.1
		R: TGTCAGCAGGGGTAAGGGTA	

Fatty acid catabolism	*ACOX1*	F: CTACAGCCTATGCCTACAAG	XM_045706191.1
		R: CTCCTGACTGACTCTCTTCT	
	*CPT1*	F: CACATCATCCCCTTCAACTG	AM230810.1
		R: CCCAAGATGATGTAGGACAC	
	*PPARα*	F: CTCTGCTGAAGTATGGAGTG	DQ294237.1
		R: CAAGTCACTGTCATCCAGC	
	*PPARβ*	F: GTATGAAGCTGGAGTACGAG	AJ416953.1
		R: CATTGAGGAAGAGGTCTACG	
	*PPARγ*	F: GTCCTACCACAGACACTTTC	AJ416951.1
		R: CCATATCACTATCGTCCAGC	

Heat shock and stress response	*HSP60*	F: CTATGCATGTTTAGGGGTGG	XM_014173566.2
		R: CAGGGTTAGCTCCTTTACTG	
	*HSP70*	F: AGACACCGAGAGACTCATC	KU885451.1
		R: CAAAGATCCCATCCTCGATG	
	*HSP90*	F: CAAGATCCTCAAGGTGATCC	NM_001173702.1
		R: CCTTCTCCACTTTCTTCTCC	
	*Serpin H1*	F: GGGATGAGAAGTTCCATGAG	XM_014163240.2
		R: GAAGATGAAGGGATGGTCAG	

Inflammation	*TNF-α*	F: GACGCTACTCAACTCTGTAC	EF079662.1
		R: CCAGGCTATGATTCCTCATC	
	*IL-1β*	F: CATGGAGAGGTTAAAGGGTG	AY617117.1
		R: GATGAACCAGTTCCTGAACC	
	*IL-6*	F: CGATATGGGAGAACGTGATC	XM_014178212.2
		R: CTCCAAGCCTGATCTAACAC	
	*MULAN*	F: GGTCATCGAGAGGTTAACTC	JF933929.1
		R: GTTCTTCTCCTTCCTCCTCT	

Apoptosis	*BAX*	F: CAGCAGTACTCAGTTGTCAG	DY722290.1
		R: CCCCTTTGTCCTTTCTTCTC	
	*Caspase-3a*	F: CAAAGAGCTAGAGGTCCAAC	DQ008070.1
		R: GTCCCACAGGATACACAATC	
	*Caspase-7*	F: CACCTCTATCACTGAGATGC	DQ008072.1
		R: GTGACCAGACTAGAATGTGG	
	*Caspase-9*	F: TATCAGGGAGAGAGACAAGG	XM_014172053.2
		R: GCCTATGATTCTGAAGGACC	
	*P53*	F: CCAGTAAGAAGACCAAGTCC	BT058777.1
		R: GTATACTGGATGTAGGGGGT	

References	*β-actin*	F: CTTCTACAACGAGCTGAGAG	AF012125.1
		R: GATACCGCAAGACTCCATAC	
	*18S*	F: CCTCGATGCTCTTAACTGAG	AJ427629.1
		R: CCCACAGAATCGAGAAAGAG	
	*EF1α*	F: GGTACATCTCAGGCTGATTG	AF321836
		R: GAAGGTGACGATCATACCAG	

Abbreviations: ACOX1, acyl‐CoA oxidase 1; BAX, bcl‐associated x protein; CPT1, carnitine palmitoyl‐transferase 1; Δ5D, delta‐5 desaturase; Δ6D, delta‐6 desaturase; ELOVL2, elongation of very long‐chain fatty acids protein 2; ELOVL5, elongation of very long‐chain fatty acids protein 5; FAS, fatty acid synthase; F, forward; HSP60, heat‐shock protein 60; HSP70, heat‐shock protein 70; HSP90, heat‐shock protein 90; IL‐1β, interleukin‐1β; IL‐6, interleukin‐6; LXR, liver X receptor; MULAN, mitochondrial ubiquitin ligase activator of NF‐κB; P53, cellular tumor antigen p53; PPARα, peroxisome proliferator‐activated receptor *α*; PPARβ, peroxisome proliferator‐activated receptor *β*; PPARγ, peroxisome proliferator‐activated receptor *γ*; R, reverse; SREBP1, sterol regulatory element‐binding protein 1; SREBP2, sterol regulatory element‐binding protein 2; TNF‐α, tumor necrosis factor‐*α*.

### 2.10. Fatty Acid Profile and Proximate Composition

Fatty acid methyl esters (FAMEs) were prepared from lipids extracted from experimental diets and muscle tissues through acid‐catalyzed transesterification. They were analyzed via gas chromatography (HP 6890, Agilent Technologies, Wilmington, DE, USA) with an HP‐88 capillary column (100 m × 250 μm × 0.25 μm, Agilent). Fatty acid profiles were expressed as percentages of the total identified FAMEs (%, w/w).

The proximate composition of the experimental diets and muscle tissues, including moisture, crude protein, crude lipid, and ash, was assessed using AOAC [26] standard methods. Moisture levels were determined after oven drying at 125°C for 3 h (OF‐22, JEIO TECH, Daejeon, South Korea). Crude protein levels were analyzed using a Kjeldahl apparatus (Kjeltec 2300, FOSS Analytical, Hillerød, Denmark). Crude lipid levels were determined using a Soxhlet extraction apparatus (SOX406, Jinan Hanon Instruments, Shandong, China). The ash content was evaluated after combustion at 550°C for 4 h using a muffle furnace (LF‐MS627, LKLAB KOREA, Namyangju, South Korea).

### 2.11. Intestinal Microbiome

Anterior and midintestinal samples were rinsed with sterile distilled water to remove residual contents and then homogenized. Microbial genomic DNA was extracted using a commercial tissue DNA extraction kit (QIAGEN, Hilden, Germany) following the manufacturer’s protocol. The V4 hypervariable region of the bacterial 16S rRNA gene was amplified using universal primers with TruSeq adapter sequences. The resulting amplicon libraries were purified, quantified, and pooled at equimolar concentrations before high‐throughput paired‐end sequencing (2 × 250 bp) on an Illumina MiSeq platform (Illumina, San Diego, CA, USA). Raw sequence data were processed using the QIIME 2 pipeline. Alpha diversity was assessed using Chao1, Shannon, and Faith’s phylogenetic diversity (PD) indices to represent microbial richness, overall diversity, and PD, respectively. Beta diversity was evaluated by principal coordinate analysis (PCoA) based on Bray–Curtis distances using MicrobiomeAnalyst (v3.0), with the resulting ordination plots visualized in two and three dimensions.

### 2.12. Statistical Analysis

Experimental diets were assigned to tanks using a completely randomized design. The tank was considered the experimental unit for all statistical analyses (*n* = 3 tanks per dietary treatment). For variables measured in individual fish, measurements from fish within each tank were averaged to obtain a single tank mean before statistical analysis. All data were analyzed using SPSS (v24.0, IBM Corp., Armonk, NY, USA). Differences among treatments were assessed using one‐way analysis of variance, followed by Tukey’s honestly significant difference test at *p*  < 0.05. Results are presented as the mean ± standard deviation. Percentage data were arcsine‐transformed prior to analysis. The optimal FO replacement level was estimated from growth performance and survival using quadratic regression analysis, following Robbins [27].

## 3. Results

### 3.1. Growth Performance, Feed Efficiency, and Survival

FBW and WG were significantly higher (*p* < 0.05) in the S20, S40, and S60 groups than in the S0 group (Table 4). SGR was significantly higher in all FO replacement groups than in the S0 group (*p* = 0.005). PER was also significantly higher in all replacement groups (*p* = 0.002), whereas FCR was significantly lower in all replacement groups (*p* = 0.001). FI did not differ significantly among dietary treatments (*p* > 0.05). Survival rates increased with increasing FO replacement levels and were significantly higher in all FO‐replaced groups than in the S0 group (*p* < 0.001; Figure 1). All growth, feed utilization, and survival parameters, except FI, exhibited significant linear and quadratic trends (*p* < 0.05). Quadratic regression analysis of WG under thermal stress indicated an optimal FO replacement level of 58.6%, whereas the estimate from the survival data under stress conditions was 81.6% (Figure 2).

**Figure 2:**
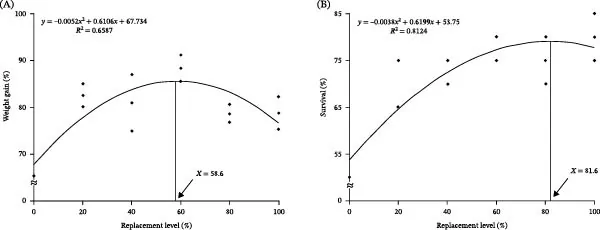
Quadratic regression analysis of weight gain (A) and survival (B) in Atlantic salmon *Salmo salar* fed diets with varying levels of fish oil replacement for 6 weeks. Abscissa: Fish oil replacement level. Ordinate: Weight gain (%) and survival (%) indicators.

**Table 4:** Growth performance, feed utilization and survival of Atlantic salmon *Salmo salar* (initial mean body weight: 101.01 ± 0.25 g) fed the experimental diets for 6 weeks under heat‐stress condition.

Diets	FBW^a^	WG^b^	SGR^c^	FI^d^	PER^e^	FCR^f^	Survival (%)
S0	165.61 ± 0.30^h^	65.49 ± 0.13^h^	1.26 ± 0.01^h^	94.17 ± 10.83	1.59 ± 0.04^h^	1.42 ± 0.03^g^	51.67 ± 2.89^i^
S20	182.47 ± 2.60^g^	82.57 ± 2.46^g^	1.51 ± 0.03^g^	106.19 ± 11.46	1.79 ± 0.01^g^	1.26 ± 0.01^h^	68.33 ± 5.77^h^
S40	181.14 ± 6.33^g^	80.94 ± 6.04^g^	1.48 ± 0.08^g^	102.61 ± 8.50	1.79 ± 0.03^g^	1.26 ± 0.02^h^	71.67 ± 2.89^gh^
S60	188.23 ± 2.28^g^	88.35 ± 2.81^g^	1.58 ± 0.04^g^	117.21 ± 12.21	1.83 ± 0.01^g^	1.23 ± 0.01^h^	78.33 ± 2.89^gh^
S80	178.74 ± 2.26^gh^	78.62 ± 1.87^gh^	1.45 ± 0.02^g^	108.50 ± 2.61	1.76 ± 0.03^g^	1.29 ± 0.02^h^	75.00 ± 5.00^gh^
S100	178.80 ± 3.43^gh^	78.80 ± 3.50^gh^	1.45 ± 0.05^g^	105.56 ± 5.56	1.75 ± 0.03^g^	1.29 ± 0.03^h^	80.00 ± 5.00^g^
Pr > *F* ^∗^							
ANOVA	0.008	0.009	0.005	0.150	0.002	0.001	<0.001
Linear	0.022	0.034	0.015	0.103	0.005	0.003	<0.001
Quadratic	0.002	0.002	0.001	0.074	<0.001	<0.001	0.006

*Note:* The experimental diets were formulated to replace fish oil in the control diet (S0) with *Schizochytrium* oil and canola oil at graded inclusion levels of 20%, 40%, 60%, 80%, and 100% (designated as S20, S40, S60, S80, and S100, respectively). Values are mean of triplicate (*n* = 3) and presented as mean ± S.D. Values with different superscripts in the same column are significantly different (*p* < 0.05).

^a^FBW, final body weight (g).

^b^WG, weight gain (%) = [(final body weight − initial body weight)/initial body weight] × 100.

^c^SGR, specific growth rate (%/d) = [(log_e_ final body weight − log_e_ initial body weight)/days] × 100.

^d^FI, feed intake (g/fish) = dry feed intake per fish, adjusted for mortality.

^e^PER, protein‐efficiency ratio = wet weight gain/total protein given.

^f^FCR, feed conversion ratio = dry feed intake/wet weight gain.

^∗^Significance probability associated with the *F*‐statistic.

### 3.2. Hematological Parameters

Plasma glucose, TG, AST, ALT, LDL, and cortisol levels were significantly lower in all FO replacement groups than in the S0 group (*p* < 0.05; Table 5). These parameters exhibited significant linear and quadratic trends (*p* < 0.05), except for ALT, which exhibited only a linear trend. CHOL levels did not differ significantly among treatments (*p* = 0.510). HDL levels were significantly higher in S60, S80, and S100 groups than in the S0 group (*p* = 0.006), with a significant linear trend observed (*p* < 0.001).

**Table 5:** Hematological parameters of Atlantic salmon *Salmo salar* fed the experimental diets for 6 weeks under heat‐stress condition.

Diets	Glucose (mg/dL)	Cholesterol (mg/dL)	Triglycerides (mg/dL)	AST (U/L)	ALT (U/L)	HDL (nmol/mL)	LDL (nmol/mL)	Cortisol (ng/mL)
S0	171.74 ± 2.53^a^	114.91 ± 1.11	277.75 ± 19.29^a^	85.26 ± 0.27^a^	46.47 ± 2.09^a^	0.61 ± 0.07^b^	2.13 ± 0.03^a^	97.93 ± 2.59^a^
S20	135.80 ± 7.64^b^	105.77 ± 8.09	229.97 ± 14.38^b^	47.69 ± 3.97^b^	30.53 ± 0.21^b^	0.87 ± 0.08^ab^	1.67 ± 0.13^b^	84.04 ± 1.24^b^
S40	134.72 ± 8.27^b^	108.37 ± 8.97	226.94 ± 3.37^b^	37.66 ± 3.19^bc^	28.33 ± 1.12^b^	0.97 ± 0.03^ab^	1.68 ± 0.04^b^	65.45 ± 2.51^c^
S60	136.53 ± 5.44^b^	115.85 ± 2.93	195.86 ± 9.45^b^	31.87 ± 3.87^c^	25.75 ± 2.20^bc^	1.10 ± 0.16^a^	1.27 ± 0.08^c^	64.83 ± 2.25^c^
S80	137.79 ± 3.08^b^	115.29 ± 3.31	191.57 ± 6.68^b^	31.55 ± 3.48^c^	22.41 ± 0.01^c^	1.16 ± 0.06^a^	1.28 ± 0.06^c^	68.67 ± 3.69^c^
S100	130.44 ± 7.06^b^	116.71 ± 5.13	203.84 ± 7.55^b^	28.96 ± 0.53^c^	10.46 ± 0.77^d^	1.18 ± 0.11^a^	1.14 ± 0.02^c^	64.17 ± 3.82^c^
Pr > *F* ^∗^								
ANOVA	0.004	0.510	0.002	<0.001	<0.001	0.006	<0.001	<0.001
Linear	0.001	0.196	<0.001	<0.001	<0.001	<0.001	<0.001	<0.001
Quadratic	0.008	0.277	0.007	<0.001	0.129	0.088	0.013	<0.001

*Note:* The experimental diets were formulated to replace fish oil in the control diet (S0) with *Schizochytrium* oil and canola oil at graded inclusion levels of 20%, 40%, 60%, 80% and 100% (designated as S20, S40, S60, S80 and S100, respectively). Values are mean of triplicate (*n* = 3) and presented as mean ± S.D. Values with different superscripts in the same column are significantly different (*p* < 0.05).

^∗^Significance probability associated with the *F*‐statistic.

### 3.3. Nonspecific Immune Responses

MPO, lysozyme, and NBT activities were significantly higher in the S40–S100 groups than in the S0 group (*p* = 0.001; Table 6). Ig levels were significantly higher in the S60–S100 groups than in the S0 and S40 groups (*p* = 0.002). These parameters exhibited significant linear trends (*p* < 0.001), whereas MPO and NBT exhibited significant quadratic trends (*p* < 0.05). Antiprotease activities did not differ significantly among treatments (*p* = 0.557).

**Table 6:** Nonspecific immunity of Atlantic salmon *Salmo salar* fed the experimental diets for 6 weeks under heat‐stress condition.

Diets	MPO (absorption)	Lysozyme (U/mL)	NBT (absorbance)	Antiprotease (% inhibition)	Ig (mg/mL)
S0	0.59 ± 0.04^c^	7.61 ± 0.17^d^	1.12 ± 0.13^c^	0.55 ± 0.01	2.04 ± 0.29^c^
S20	1.39 ± 0.04^bc^	7.98 ± 0.02^cd^	1.45 ± 0.07^bc^	0.58 ± 0.04	2.54 ± 0.18^bc^
S40	1.74 ± 0.03^ab^	8.59 ± 0.39^bc^	1.62 ± 0.11^b^	0.56 ± 0.01	2.25 ± 0.82^c^
S60	2.51 ± 0.22^a^	8.73 ± 0.09^abc^	1.85 ± 0.15^ab^	0.57 ± 0.01	4.54 ± 0.29^a^
S80	2.52 ± 0.36^a^	9.45 ± 0.23^a^	2.11 ± 0.06^a^	0.58 ± 0.01	4.38 ± 0.41^a^
S100	2.02 ± 0.40^ab^	9.26 ± 0.13^ab^	1.79 ± 0.11^ab^	0.57 ± 0.01	4.17 ± 0.12^ab^
Pr > *F* ^∗^					
ANOVA	0.001	0.001	0.001	0.557	0.002
Linear	<0.001	<0.001	<0.001	0.467	<0.001
Quadratic	0.002	0.125	0.007	0.635	0.307

*Note:* The experimental diets were formulated to replace fish oil in the control diet (S0) with *Schizochytrium* oil and canola oil at graded inclusion levels of 20%, 40%, 60%, 80%, and 100% (designated as S20, S40, S60, S80, and S100, respectively). Values are mean of triplicate (*n* = 3) and presented as mean ± S.D. Values with different superscripts in the same column are significantly different (*p* < 0.05).

^∗^Significance probability associated with the *F*‐statistic.

### 3.4. Antioxidant Capacity

SOD, GPx, CAT, and TAOC activities were significantly higher in all FO replacement groups than in the S0 group (*p* < 0.001), showing significant linear and quadratic trends (*p* < 0.001; Table 7). In contrast, MDA levels were significantly lower in all FO‐replaced groups than in the S0 group (*p* < 0.001), with significant linear and quadratic trends observed (*p* < 0.001).

**Table 7:** Antioxidant capacity of Atlantic salmon *Salmo salar* fed the experimental diets for 6 weeks under heat‐stress condition.

Diets	SOD (% inhibition)	GPx (mU/mL)	CAT (mU/mL)	MDA (nmol/mg)	TAOC (μmol/L)
S0	74.06 ± 0.62^e^	687.67 ± 17.92^c^	170.61 ± 0.84^b^	2.25 ± 0.17^a^	534.67 ± 8.58^e^
S20	84.00 ± 1.77^d^	781.14 ± 10.84^b^	195.94 ± 0.88^a^	1.55 ± 0.17^b^	569.50 ± 9.58^d^
S40	89.49 ± 1.99^c^	936.50 ± 18.19^a^	198.57 ± 4.56^a^	1.46 ± 0.15^b^	791.83 ± 1.26^c^
S60	92.07 ± 1.44^bc^	958.91 ± 11.67^a^	196.50 ± 8.05^a^	1.46 ± 0.02^b^	807.33 ± 8.52^bc^
S80	94.13 ± 0.92^ab^	974.45 ± 11.44^a^	204.21 ± 6.97^a^	1.47 ± 0.10^b^	824.67 ± 3.01^ab^
S100	96.74 ± 1.47^a^	972.67 ± 22.11^a^	196.65 ± 1.86^a^	1.69 ± 0.07^b^	841.00 ± 6.93^a^
Pr > *F* ^∗^					
ANOVA	<0.001	<0.001	<0.001	<0.001	<0.001
Linear	<0.001	<0.001	<0.001	<0.001	<0.001
Quadratic	<0.001	<0.001	<0.001	<0.001	<0.001

*Note:* The experimental diets were formulated to replace fish oil in the control diet (S0) with *Schizochytrium* oil and canola oil at graded inclusion levels of 20%, 40%, 60%, 80%, and 100% (designated as S20, S40, S60, S80, and S100, respectively). Values are mean of triplicate (*n* = 3) and presented as mean ± S.D. Values with different superscripts in the same column are significantly different (*p* < 0.05).

^∗^Significance probability associated with the *F*‐statistic.

### 3.5. Digestive Enzyme Activity and Fatty Acid *β*‐Oxidation

Trypsin activity was significantly higher in the S40–S100 groups than in the S0 group (*p* = 0.001), whereas chymotrypsin activity did not differ significantly among treatments (*p* = 0.089; Table 8). Lipase activity was significantly higher in all FO replacement groups than in the S0 group (*p* < 0.001). Pepsin activity was significantly elevated in the S60 group compared with that in the S0 group (*p* = 0.030). *β*‐Oxidation activity was significantly higher in the S40–S100 groups than in the S0 group (*p* < 0.001). Significant linear trends were observed for trypsin, lipase, and *β*‐oxidation (*p* < 0.001) and significant quadratic trends (*p* < 0.05) were observed for all parameters except chymotrypsin.

**Table 8:** Digestive enzyme and *β*‐oxidation activities of Atlantic salmon *Salmo salar* fed the experimental diets for 6 weeks under heat‐stress condition.

Diets	Intestine			Stomach	Liver
	Trypsin (U/protein)	Chymotrypsin (U/protein)	Lipase (U/protein)	Pepsin (U/protein)	*β*‐Oxidation (μmol/L × min)
S0	1.89 ± 0.04^d^	1.04 ± 0.04	63.78 ± 3.72^c^	36.43 ± 0.77^b^	0.41 ± 0.03^c^
S20	3.14 ± 0.19^cd^	1.21 ± 0.02	124.77 ± 3.98^ab^	42.12 ± 2.18^ab^	0.66 ± 0.09^bc^
S40	4.04 ± 0.13^bc^	1.19 ± 0.14	121.39 ± 5.86^b^	39.79 ± 1.85^ab^	0.85 ± 0.09^ab^
S60	5.22 ± 0.71^ab^	1.18 ± 0.02	134.33 ± 7.49^ab^	45.52 ± 3.06^a^	1.07 ± 0.11^a^
S80	6.41 ± 0.87^a^	1.20 ± 0.07	149.78 ± 9.15^a^	44.19 ± 0.42^ab^	0.98 ± 0.15^a^
S100	4.46 ± 0.24^bc^	1.03 ± 0.00	136.12 ± 8.62^ab^	39.54 ± 2.40^ab^	1.08 ± 0.16^a^
Pr > *F* ^∗^					
ANOVA	0.001	0.089	<0.001	0.030	<0.001
Linear	<0.001	0.827	<0.001	0.060	<0.001
Quadratic	0.003	0.100	0.001	0.010	0.009

*Note:* The experimental diets were formulated to replace fish oil in the control diet (S0) with *Schizochytrium* oil and canola oil at graded inclusion levels of 20%, 40%, 60%, 80%, and 100% (designated as S20, S40, S60, S80, and S100, respectively). Values are mean of triplicate (*n* = 3) and presented as mean ± S.D. Values with different superscripts in the same column are significantly different (*p* < 0.05).

^∗^Significance probability associated with the *F*‐statistic.

### 3.6. Histopathological Analysis

Compared with the S0 group, villi length was significantly increased in all FO replacement groups (*p* < 0.001; Table 9). Villi width was significantly higher in the S40–S100 groups than in the S0 group (*p* = 0.003). The number of vacuoles was significantly lower in all replacement groups than in the S0 group (*p* < 0.001). All histopathological parameters exhibited significant linear and quadratic trends (*p* < 0.05).

**Table 9:** Intestine histomorphology of Atlantic salmon *Salmo salar* fed the experimental diets for 6 weeks under heat‐stress condition.

Diets	Villi length (µm)	Villi width (µm)	Vacuolization (counts/villi)
S0	281.67 ± 17.56^c^	34.02 ± 4.58^b^	13.57 ± 0.81^a^
S20	331.67 ± 7.64^b^	41.39 ± 0.53^ab^	8.32 ± 1.73^b^
S40	384.80 ± 16.04^a^	45.03 ± 3.61^a^	7.79 ± 0.37^bc^
S60	415.00 ± 13.23^a^	46.70 ± 1.15^a^	7.61 ± 0.62^bc^
S80	402.71 ± 16.17^a^	46.22 ± 4.01^a^	6.00 ± 0.50^bc^
S100	405.02 ± 5.00^a^	44.71 ± 3.06^a^	5.77 ± 0.67^c^
Pr > *F* ^∗^			
ANOVA	<0.001	0.003	<0.001
Linear	<0.001	0.001	<0.001
Quadratic	<0.001	0.004	0.001

*Note:* The experimental diets were formulated to replace fish oil in the control diet (S0) with *Schizochytrium* oil and canola oil at graded inclusion levels of 20%, 40%, 60%, 80%, and 100% (designated as S20, S40, S60, S80, and S100, respectively). Values are mean of triplicate (*n* = 3) and presented as mean ± S.D. Values with different superscripts in the same column are significantly different (*p* < 0.05).

^∗^Significance probability associated with the *F*‐statistic.

### 3.7. Gene Expression

The results of the expression of genes involved in fatty acid desaturation and elongation are presented in Figure 3. The expression of *FAS*, Δ*5D*, and Δ*6D* was significantly downregulated in all FO replacement groups compared with that in the S0 group (*p* < 0.001). Similarly, *ELOVL2* and *ELOVL5* expression was significantly downregulated in the S40–S100 groups compared with that in the S0 group (*p* ≤ 0.001). Significant linear and quadratic trends were observed for all genes except *ELOVL2*, which showed only a significant linear trend (*p* < 0.05).

**Figure 3:**
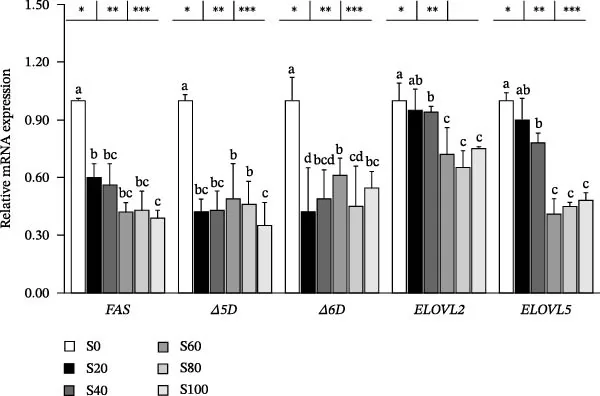
Relative mRNA expression levels of fatty acid desaturation and elongation‐related genes in Atlantic salmon *Salmo salar* fed experimental diets under heat‐stress conditions for 6 weeks. Gene expression levels of fatty acid synthase (*FAS*), delta‐5 desaturase (Δ*5D*), delta‐6 desaturase (Δ*6D*), elongation of very long‐chain fatty acids protein 2 (*ELOVL2*), and *ELOVL5* were analyzed. Values are presented as means of triplicate groups (*n* = 3). Different lowercase letters above the bars indicate significant differences among dietary treatments (*p* < 0.05). Asterisks denote overall statistical significance and trend analysis:  ^∗^ indicates a significant effect by one‐way ANOVA (*p* < 0.05),  ^∗∗^ indicates a significant linear trend, and  ^∗∗∗^ indicates a significant quadratic trend.

The results of the expression of genes associated with fatty acid transcription factors are presented in Figure 4. *LXR*, *SREBP1*, and *SREBP2* expression levels were significantly downregulated in all FO replacement groups compared with those in the S0 group (*p* < 0.001) and all parameters exhibited significant linear and quadratic trends (*p* < 0.05).

**Figure 4:**
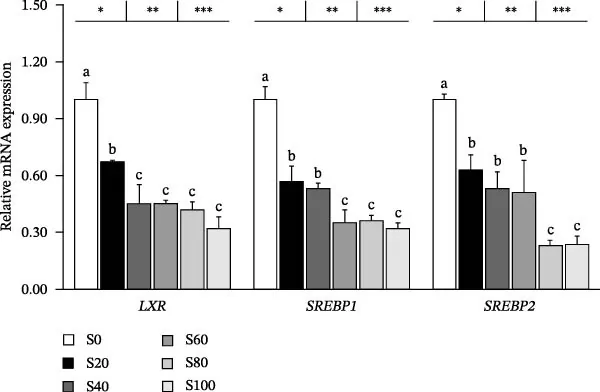
Relative mRNA expression levels of fatty acid transcription factor‐related genes in Atlantic salmon *Salmo salar* fed experimental diets under heat‐stress conditions for 6 weeks. Gene expression levels of liver X receptor (*LXR*), sterol regulatory element‐binding protein 1 (*SREBP1*), and *SREBP2* were analyzed. Values are presented as means of triplicate groups (*n* = 3). Different lowercase letters above the bars indicate significant differences among dietary treatments (*p* < 0.05). Asterisks denote overall statistical significance and trend analysis:  ^∗^ indicates a significant effect by one‐way ANOVA (*p* < 0.05),  ^∗∗^ indicates a significant linear trend, and  ^∗∗∗^ indicates a significant quadratic trend.

Fatty acid catabolism‐related gene expression findings are presented in Figure 5. Compared with the S0‐group expression, *ACOX1*, *CPT1*, *PPARα*, and *PPARβ* expression levels were significantly upregulated in all FO replacement groups (*p* < 0.001). *PPARγ* expression was significantly higher in the S40–S100 groups than in the S0 group (*p* < 0.001). All parameters exhibited significant linear and quadratic trends (*p* < 0.05).

**Figure 5:**
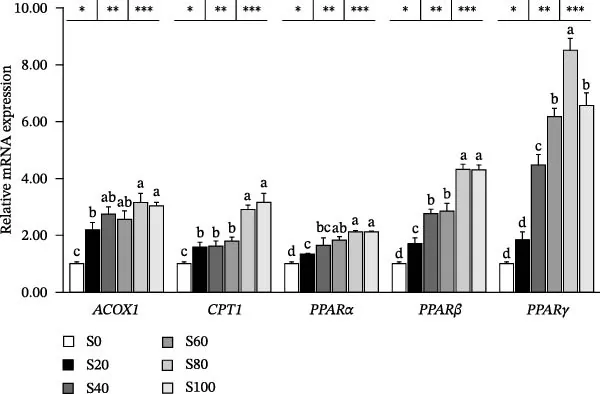
Relative mRNA expression levels of fatty acid catabolism‐related genes in Atlantic salmon *Salmo salar* fed experimental diets under heat‐stress conditions for 6 weeks. Gene expression levels of acyl‐CoA oxidase 1 (*ACOX1*), carnitine palmitoyl‐transferase 1 (*CPT1*), peroxisome proliferator‐activated receptor *α* (*PPARα*), *PPARβ*, and *PPARγ* were analyzed. Values are presented as means of triplicate groups (*n* = 3). Different lowercase letters above the bars indicate significant differences among dietary treatments (*p* < 0.05). Asterisks denote overall statistical significance and trend analysis:  ^∗^ indicates a significant effect by one‐way ANOVA (*p* < 0.05),  ^∗∗^ indicates a significant linear trend, and  ^∗∗∗^ indicates a significant quadratic trend.

The expression of genes associated with heat shock and stress responses is shown in Figure 6. *HSP60*, *HSP70*, and *HSP90* expression levels were significantly downregulated in all FO replacement groups compared with those in the S0 group (*p* < 0.001). In contrast, *Serpin H1* expression was significantly lower in the S60–S100 groups than in the S0–S40 groups (*p* < 0.001). All parameters exhibited significant linear and quadratic trends (*p* < 0.05).

**Figure 6:**
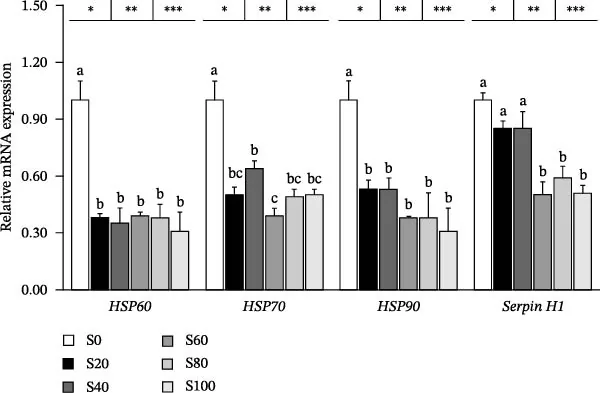
Relative mRNA expression levels of heat shock and stress responses‐related genes in Atlantic salmon *Salmo salar* fed experimental diets under heat‐stress conditions for 6 weeks. Gene expression levels of heat‐shock protein 60 (*HSP60*), *HSP70*, *HSP90*, and *Serpin H1* were analyzed. Values are presented as means of triplicate groups (*n* = 3). Different lowercase letters above the bars indicate significant differences among dietary treatments (*p* < 0.05). Asterisks denote overall statistical significance and trend analysis:  ^∗^ indicates a significant effect by one‐way ANOVA (*p* < 0.05),  ^∗∗^ indicates a significant linear trend, and  ^∗∗∗^ indicates a significant quadratic trend.

Inflammation‐related gene expression analysis is presented in Figure 7. *TNF-α* expression was significantly downregulated in the S60–S100 groups compared with that in the S0–S40 groups (*p* < 0.001), exhibiting a significant linear trend (*p* < 0.001). *IL-1β*, *IL-6*, and *MULAN* expression levels were significantly decreased (*p* < 0.001) in all FO replacement groups compared with those in the S0 group, with significant linear and quadratic trends observed (*p* < 0.05).

**Figure 7:**
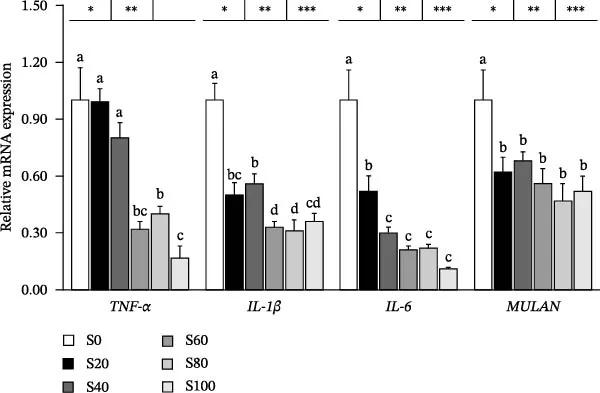
Relative mRNA expression levels of inflammation‐related genes in Atlantic salmon *Salmo salar* fed experimental diets under heat‐stress conditions for 6 weeks. Gene expression levels of tumor necrosis factor‐*α* (*TNF-α*), interleukin‐1β (*IL-1β*), interleukin 6 (*IL-6*), and mitochondrial ubiquitin ligase activator of NF‐κB (*MULAN*) were analyzed. Values are presented as means of triplicate groups (*n* = 3). Different lowercase letters above the bars indicate significant differences among dietary treatments (*p* < 0.05). Asterisks denote overall statistical significance and trend analysis:  ^∗^ indicates a significant effect by one‐way ANOVA (*p* < 0.05),  ^∗∗^ indicates a significant linear trend, and  ^∗∗∗^ indicates a significant quadratic trend.

Apoptosis‐related gene expression results are shown in Figure 8. *BAX* and *Caspase-3a* expression was significantly downregulated in the S40–S100 groups compared with that in the S0 group (*p* < 0.05), with significant linear and quadratic trends (*p* < 0.05). Similarly, *Caspase-7*, *Caspase-9*, and *P53* expression was significantly lower in all FO replacement groups than in the S0 group (*p* < 0.001), with significant linear and quadratic trends observed (*p* < 0.05).

**Figure 8:**
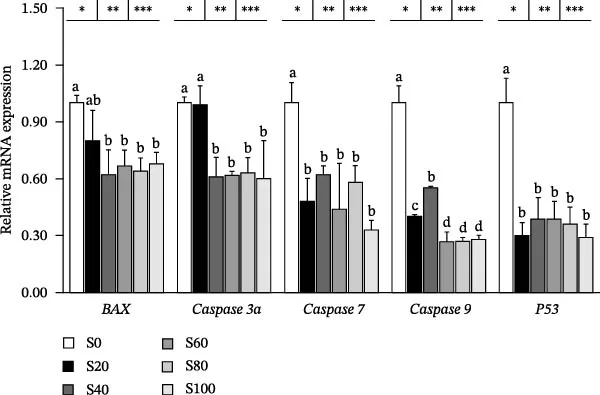
Relative mRNA expression levels of apoptosis‐related genes in Atlantic salmon *Salmo salar* fed experimental diets under heat‐stress conditions for 6 weeks. Gene expression levels of bcl‐associated x protein (*BAX*), *Caspase-3a*, *Caspase-7*, *Caspase-9*, and cellular tumor antigen p53 (*P53*) were analyzed. Values are presented as means of triplicate groups (*n* = 3). Different lowercase letters above the bars indicate significant differences among dietary treatments (*p* < 0.05). Asterisks denote overall statistical significance and trend analysis:  ^∗^ indicates a significant effect by one‐way ANOVA (*p* < 0.05),  ^∗∗^ indicates a significant linear trend, and  ^∗∗∗^ indicates a significant quadratic trend.

### 3.8. Fatty Acid Profile and Proximate Composition

No significant differences were observed in muscle C14:0, C16:0, C16:1, C18:0, C18:1n9, and C20:4n6 levels among treatments (*p* > 0.05; Table 10). LA and C22:5n6 levels were significantly higher in the S60–S100 groups than in the S0 group (*p* < 0.05), whereas ALA levels were significantly lower in the S80 and S100 groups than in the S0 group (*p* = 0.008). EPA levels were significantly lower in the S60–S100 groups than in the S0 group (*p* = 0.003). In contrast, DHA levels were significantly higher in all FO replacement groups than in the S0 group (*p* < 0.001). Muscle ∑*n*−3 fatty acid levels were significantly higher in the S40–S100 groups than in the S0 group (*p* < 0.001), while ∑*n*−6 fatty acid levels were significantly higher in the S80 and S100 groups than in the S0 group (*p* = 0.003). Significant linear trends were observed for LA, ALA, C22:5n6, EPA, ∑*n*‐3, and ∑*n*‐6, whereas significant linear and quadratic trends were observed for DHA (*p* < 0.05).

**Table 10:** Fatty acid profile (% of total FAME) in muscle of Atlantic salmon *Salmo salar* fed the experimental diets for 6 weeks under heat‐stress condition.

Fatty acids	Experimental diets						Pr > *F* ^∗^		
	S0	S20	S40	S60	S80	S100	ANOVA	Linear	Quadratic
SFA^a^									
C14:0	2.69 ± 0.01	2.61 ± 0.01	2.62 ± 0.02	2.61 ± 0.06	2.48 ± 0.11	2.54 ± 0.20	0.471	0.100	0.847
C16:0	18.04 ± 0.01	17.99 ± 0.03	17.50 ± 0.65	17.41 ± 0.28	17.50 ± 0.26	17.49 ± 0.16	0.361	0.086	0.268
C18:0	6.26 ± 0.14	6.19 ± 0.05	6.14 ± 0.09	6.24 ± 0.13	6.19 ± 0.10	6.44 ± 0.12	0.249	0.182	0.069
MUFA^b^									
C16:1	3.80 ± 0.01	3.71 ± 0.02	3.73 ± 0.04	3.70 ± 0.01	3.68 ± 0.03	3.73 ± 0.10	0.343	0.147	0.158
C18:1n9	32.21 ± 0.08	32.32 ± 0.02	31.49 ± 0.62	31.60 ± 0.51	31.21 ± 0.21	31.19 ± 1.44	0.512	0.094	0.763
PUFA^c^									
C18:2n6	18.31 ± 0.02^f^	18.89 ± 0.05^ef^	19.02 ± 0.08^ef^	19.28 ± 0.16^e^	19.81 ± 0.49^de^	20.30 ± 0.01^d^	0.001	<0.001	0.836
C18:3n3	7.46 ± 0.01^d^	7.02 ± 0.01^de^	6.78 ± 0.04^def^	6.70 ± 0.15^def^	6.41 ± 0.36^ef^	6.24 ± 0.26^f^	0.008	<0.001	0.481
C20:4n6	1.14 ± 0.11	1.09 ± 0.19	1.13 ± 0.11	1.11 ± 0.15	1.28 ± 0.11	1.10 ± 0.11	0.718	0.638	0.903
C20:5n3	2.18 ± 0.05^d^	2.09 ± 0.02^def^	2.10 ± 0.01^de^	2.08 ± 0.03^ef^	2.02 ± 0.01^ef^	1.99 ± 0.01^f^	0.003	<0.001	0.841
C22:5n6	0.03 ± 0.01^e^	0.07 ± 0.03^de^	0.07 ± 0.02^de^	0.09 ± 0.02^d^	0.12 ± 0.03^d^	0.12 ± 0.03^d^	0.002	<0.001	0.603
C22:6n3	8.18 ± 0.04^h^	9.22 ± 0.05^g^	10.20 ± 0.07^f^	11.18 ± 0.04^e^	12.47 ± 0.07^d^	12.58 ± 0.10^d^	<0.001	<0.001	<0.001
∑*n*‐3	17.82 ± 0.08^g^	18.32 ± 0.02^fg^	19.08 ± 0.12^f^	19.96 ± 0.08^e^	20.89 ± 0.42^d^	20.81 ± 0.15^d^	<0.001	<0.001	0.126
∑*n*‐6	19.44 ± 0.11^f^	20.08 ± 0.25^ef^	20.23 ± 0.03^ef^	20.54 ± 0.29^def^	21.18 ± 0.58^de^	21.49 ± 0.08^d^	0.003	<0.001	0.879

*Note:* The experimental diets were formulated to replace fish oil in the control diet (S0) with *Schizochytrium* oil and canola oil at graded inclusion levels of 20%, 40%, 60%, 80%, and 100% (designated as S20, S40, S60, S80, and S100, respectively). Values are mean of triplicate (*n* = 3) and presented as mean ± S.D. Values with different superscripts in the same column are significantly different (*p* < 0.05).

^a^Saturated fatty acids.

^b^Monounsaturated fatty acids.

^c^Polyunsaturated fatty acids.

^∗^Significance probability associated with the *F*‐statistic.

No significant differences were observed among treatments in terms of muscle moisture content, crude lipid levels, or ash content (*p* > 0.05) (Table 11). However, the crude protein content was significantly higher in the S40–S100 groups than in the S0 group (*p* = 0.003), with significant linear and quadratic trends (*p* < 0.05).

**Table 11:** Proximate composition (% wet basis) in muscle of Atlantic salmon *Salmo salar* fed the experimental diets for 6 weeks under heat‐stress condition.

Diets	Moisture	Crude protein	Crude lipid	Ash
S0	68.37 ± 1.22	21.17 ± 0.77^b^	7.29 ± 0.33	1.89 ± 0.32
S20	69.21 ± 0.86	22.12 ± 0.17^b^	8.03 ± 0.34	1.76 ± 0.02
S40	68.27 ± 0.80	24.18 ± 0.57^a^	7.85 ± 0.04	1.77 ± 0.04
S60	67.86 ± 0.91	24.31 ± 0.53^a^	7.82 ± 0.09	1.84 ± 0.05
S80	68.66 ± 1.61	24.29 ± 0.12^a^	7.88 ± 0.21	1.79 ± 0.00
S100	69.32 ± 2.55	24.21 ± 0.55^a^	7.52 ± 0.48	1.58 ± 0.01
Pr > *F* ^∗^				
ANOVA	0.799	0.003	0.263	0.397
Linear	0.698	<0.001	0.712	0.132
Quadratic	0.441	0.013	0.055	0.493

*Note:* The experimental diets were formulated to replace fish oil in the control diet (S0) with *Schizochytrium* oil and canola oil at graded inclusion levels of 20%, 40%, 60%, 80%, and 100% (designated as S20, S40, S60, S80, and S100, respectively). Values are mean of triplicate (*n* = 3) and presented as mean ± S.D. Values with different superscripts in the same column are significantly different (*p* < 0.05).

^∗^Significance probability associated with the *F*‐statistic.

### 3.9. Intestinal Microbiome

At the family level, relative abundance analysis identified Vibrionaceae, Muribaculaceae, Lachnospiraceae, Lactobacillaceae, and Moraxellaceae as the five most dominant families across treatments (Figure 9). The S0 group exhibited a markedly higher abundance of Vibrionaceae (56.99%) than the replacement groups (6.98%–17.60%). In contrast, Lachnospiraceae abundance was lower in the S0 (5.80%) group than in all other groups (14.43%–18.22%).

**Figure 9:**
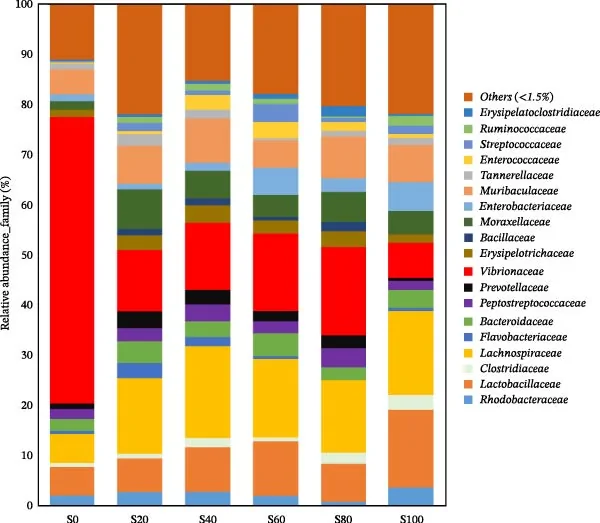
Relative abundance of intestinal microbiota at the family level in Atlantic salmon (*Salmo salar*) fed experimental diets for 6 weeks under heat‐stress conditions. Families with a relative abundance of <1.5% were grouped and presented as “Others”.

At the genus level, *Vibrio*, *Lactobacillus*, *Muribaculaceae*, *Acinetobacter*, and *Bacteroides* were the predominant taxa across treatments (Figure 10). Notably, *Bacillus*, *Aliivibrio*, and *Turicibacter* were not detected in the S0 group. The relative abundance of *Lactobacillus*, *Bacillus*, and *Enterococcus* increased with increasing dietary inclusion level of SCZO. In contrast, the relative abundance of *Vibrio* was highest in the S0 group (14.53%) and was markedly lower in the replacement groups (0.70%–3.97%). Similarly, *Photobacterium* (4.85%) and *Streptococcus* (6.28%) were more abundant in the S0 group than in the other treatment groups (0.016%–0.85% and 0.69%–1.56%, respectively).

**Figure 10:**
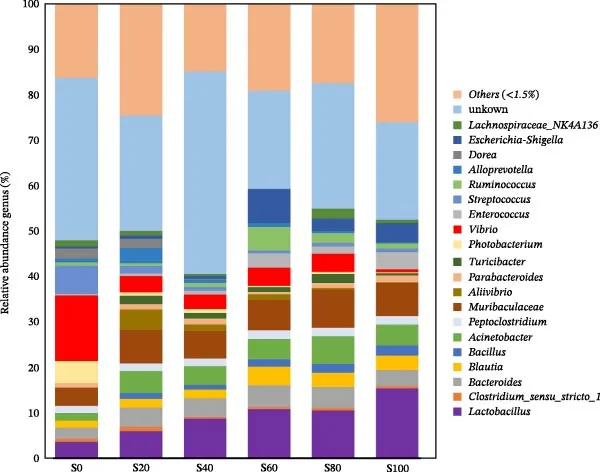
Relative abundance of intestinal microbiota at the genus level in Atlantic salmon *Salmo salar* fed experimental diets for 6 weeks under heat‐stress conditions. Genera with a relative abundance of <1.5% were grouped and presented as “Others”.

Alpha‐diversity indices are shown in Figure 11. Chao1, Shannon and Faith’s PD indices increased with increasing levels of FO replacement by SCZO combined with CNO, exhibiting significant linear trends (*p* < 0.001).

**Figure 11:**
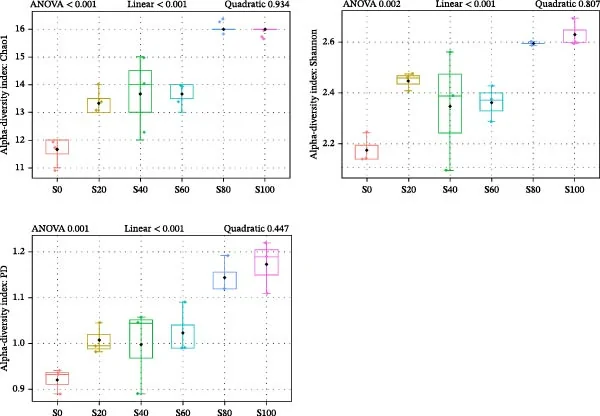
Alpha‐diversity indices (Chao1, Shannon and Faith’s PD) of the intestinal microbiota in Atlantic salmon *Salmo salar* after 6 weeks of feeding experimental diets under heat‐stress conditions. Data represent three replicate tanks per dietary treatment (*n* = 3). Individual points represent tank‐level values. Black points indicate the mean, horizontal lines within boxes indicate the median, boxes represent the interquartile range (25th–75th percentiles), and whiskers indicate the minimum and maximum values.

Beta‐diversity of the intestinal microbiota was evaluated using PCoA (Figure 12). Distinct clustering was observed among the dietary groups, indicating that replacement of FO with SCZO combined with CNO altered the intestinal microbial community structure. The S0 group clustered separately from the replacement groups along PC1, suggesting a shift in microbial composition following dietary substitution. Notably, the S80 and S100 groups were more distinct from the other treatments, indicating greater dissimilarity in microbial communities. In contrast, the partial replacement groups (S20–S60) clustered more closely, suggesting relatively similar microbial profiles.

**Figure 12:**
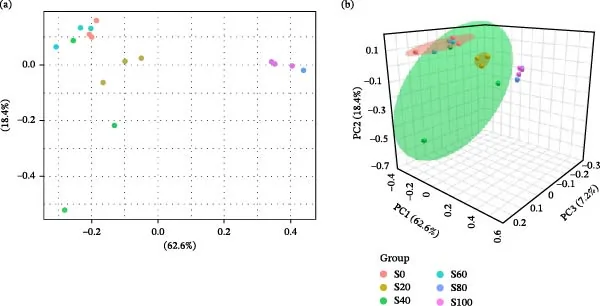
Beta diversity of intestinal microbiota in Atlantic salmon *Salmo salar* fed experimental diets for 6 weeks under heat‐stress conditions. (a) Two‐dimensional ordination plot based on principal coordinate analysis (PCoA). (b) Three‐dimensional (3D) PCoA plot showing sample clustering patterns. The first three principal coordinates explained 62.6% (PC1), 18.4% (PC2), and 7.2% (PC3) of the total variation, respectively.

## 4. Discussion

Growth performance, feed efficiency, and survival were significantly improved in fish fed diets in which FO was replaced with SCZO combined with CNO, particularly under elevated temperature conditions. Previous studies have shown that replacing FO with SCZ‐derived products, including SCZO or SCZ powder, does not adversely affect the growth, feed utilization, or survival of *S. salar* under normal rearing conditions [16, 28]. However, the present study was conducted under thermal stress, a condition known to substantially alter metabolic demands and physiological responses [13]. Under high‐temperature conditions, fish experience increased metabolic rates, elevated oxidative stress, and impaired membrane stability, collectively increasing the demand for essential fatty acids such as EPA and DHA [4, 14]. Reported dietary EPA + DHA requirements for *S. salar* vary depending on life stage, dietary composition, and rearing conditions. Rosenlund et al. [29] suggested that ~10 g/kg dietary EPA + DHA is required to support optimal long‐term growth during the seawater phase, while Bou et al. [30] reported beneficial effects of 17 g/kg EPA + DHA under challenging environmental conditions. In the present study, dietary EPA + DHA remained relatively constant at 24.4–24.9 g/kg across all treatments and therefore exceeded these previously reported levels. Notably, the CLO used in the present study contained relatively less DHA than EPA, resulting in dietary levels of 8.9 g/kg DHA and 15.7 g/kg EPA in S0. This characteristic of the CLO was considered during diet formulation, and its inclusion level was adjusted to ensure that the total dietary EPA + DHA supply met previously reported requirements for *S. salar*. However, the results showed that SCZO‐based diets resulted in superior growth, improved feed efficiency, and higher survival compared with those in the S0 group. Because total dietary EPA + DHA remained comparable among treatments, these responses are unlikely to be attributable to inadequate total EPA + DHA supply in S0. Instead, they occurred concurrently with a progressive increase in dietary DHA and decrease in EPA as CLO was replaced by SCZO and CNO. These findings suggest that the increased dietary DHA supply may have contributed to physiological stability and survival despite the concurrent decrease in dietary EPA levels [31]. Notably, the S100 group exhibited the highest survival, which coincided with the highest dietary DHA level [32]. It should also be considered that CLO may differ from other lipid sources in characteristics beyond its fatty acid composition. The PV, *p*‐AV, and TOTOX values determined at the time of diet preparation were within the limits specified by the Codex Standard for Fish Oils (CXS 329‐2017) and did not indicate greater oxidative deterioration of the CLO relative to the other lipid sources (Table S1). Nevertheless, the possibility that the lipid sources contributed different amounts of naturally occurring fat‐soluble vitamins cannot be excluded as an additional factor influencing the observed responses.

DHA is crucial in maintaining membrane fluidity and integrity, particularly under high‐temperature stress, in which elevated temperatures can disrupt lipid bilayers, thereby enhancing the survival of *S. salar* [30]. DHA modulates the function of membrane‐bound proteins, including ion channels and receptors, thereby supporting cellular homeostasis [33]. Although dietary EPA levels decreased with increasing FO replacement levels, stress‐related functions may have been sufficiently supported by the combined EPA + DHA supply and the elevated DHA availability in SCZO‐containing diets. DHA has been reported to play a more critical role than EPA in maintaining membrane stability, mitochondrial function, and stress tolerance under elevated temperature conditions [30]. Compared with EPA, DHA has a higher degree of unsaturation and is more readily incorporated into membrane phospholipids, contributing more effectively to membrane fluidity and cellular resilience during thermal stress [34]. EPA can be partly used for energy and eicosanoid production, whereas DHA is more likely to be retained in structural lipids to preserve membrane function under stress [35]. Overall, the findings indicate the importance of dietary DHA in supporting growth and survival under thermal stress, consistent with previous studies highlighting SCZ as a promising alternative source of *n*‐3 LC‐PUFAs [11]. In addition, because the CLO used as the control lipid source may not be representative of the FO predominantly used in contemporary commercial salmon feeds, validation using other commercially relevant FOs would be warranted before broadly extrapolating the present findings to industrial production. It should also be noted that the experimental diets were produced using a laboratory‐scale compression pellet mill without an extrusion process. Because extrusion can influence the physical properties, water stability, and nutrient utilization of aquafeeds, further validation using commercially extruded diets is warranted before direct application of the present replacement strategy under industrial farming conditions.

High‐temperature stress impairs intestinal function by impairing epithelial integrity, reducing digestive enzyme secretion, and increasing oxidative damage, which ultimately reduces nutrient utilization efficiency in fish [36, 37]. In the present study, replacing FO with SCZO and CNO significantly enhanced the activities of digestive enzymes, including trypsin, lipase, and pepsin, indicating improved digestive capacity. These findings are consistent with previous studies showing that dietary inclusion of SCZO or SCZ powder improved the digestibility of protein, PUFA, and DHA in salmonids, suggesting enhanced nutrient utilization efficiency [9, 11, 15]. In addition, increased activities of digestive enzymes such as amylase and lipase have been reported in golden pompano (*Trachinotus ovatus*) fed SCZ‐based diets, indicating a stimulatory effect on the digestive function [38]. These effects are closely linked to dietary lipid composition, as fatty acids directly influence enterocyte membrane dynamics, controlling enzyme synthesis, secretion, and nutrient transport [39]. In particular, the high DHA content of the diet likely contributed to improved enterocyte membrane fluidity and flexibility, thereby facilitating vesicular trafficking, enzyme secretion, and nutrient transport under stress conditions [40]. Concomitantly, intestinal morphology significantly improved, with longer and wider villi and reduced vacuolation. These morphological responses are consistent with the importance of dietary HUFA supply for maintaining intestinal integrity and resilience in *S. salar*, particularly under chronic stress conditions [40]. Under high‐temperature stress, excessive reactive oxygen species (ROS) accumulation can disrupt mitochondrial function and induce epithelial damage, resulting in vacuolization and impaired absorption [41]. In this context, DHA plays a protective role by stabilizing mitochondrial membranes and reducing oxidative damage, preserving enterocyte viability, and supporting epithelial turnover [42]. These morphological improvements suggest that dietary lipid replacement alleviates oxidative damage and maintains intestinal structural integrity. In parallel, enhanced lipase activity indicates improved lipid digestion and nutrient absorption, which may support enterocyte proliferation and intestinal epithelial renewal [43], a crucial factor under high‐temperature stress when energy demands increase for cellular maintenance and repair. These mechanisms provide a physiological explanation for the improved growth and survival of *S. salar* under high‐temperature stress.

High‐temperature exposure can induce substantial hepatic stress and metabolic disturbance in *S. salar*, including alterations in heat‐shock, oxidative‐stress, and metabolic pathways [44]. The present study showed that replacing FO with SCZO and CNO significantly reduced plasma glucose, TG, AST, ALT, LDL, and cortisol levels while increasing HDL levels. These changes indicate improved metabolic regulation and physiological stability. Elevated plasma glucose is a typical stress response mediated by cortisol‐induced gluconeogenesis [45]. Therefore, the concurrent reductions in plasma glucose and cortisol levels suggest that dietary SCZO may have attenuated stress‐induced endocrine and metabolic disturbances under high‐temperature conditions. Similarly, the lower plasma AST and ALT activities observed in the FO‐replaced groups may indicate better maintenance of hepatic integrity as elevated AST and ALT activities have been associated with hepatic dysfunction in *S. salar* exposed to high temperatures [46]. The reduction in TG and LDL levels along with the increase in HDL suggests enhanced lipid transport and utilization efficiency, which are critical for maintaining energy homeostasis under stressful conditions [47]. These effects are likely attributable to the high DHA level of SCZO, which plays an important role in modulating membrane fluidity, improving lipoprotein metabolism, and regulating metabolic pathways [33]. In particular, DHA has been reported to suppress lipogenesis while promoting lipid oxidation through the regulation of transcription factors such as *SREBP* and *PPAR*, thereby contributing to an improved metabolic balance [48]. Consistent with these findings, Lee et al. [49] reported that replacing FO with a combination of SCZO and soybean oil improved hematological parameters in rainbow trout (*Oncorhynchus mykiss*), supporting the beneficial effects of microalgae‐based lipid sources on metabolic health. Collectively, these results indicate that replacing FO with the combination of SCZO and CNO enhances metabolic homeostasis and physiological stability in *S. salar* under high‐temperature stress, which may have contributed to the improved growth performance and survival observed in the present study.

High‐temperature stress impairs immune function and induces excessive ROS generation, causing oxidative damage and reducing disease resistance in fish [37]. In the present study, elevated MPO, lysozyme, and NBT activities as well as increased Ig levels in the FO replacement groups indicate an enhanced nonspecific immune response. Concurrently, antioxidant capacity improved markedly, as evidenced by increased SOD, GPx, CAT, and TAOC activities as well as decreased MDA levels, indicating reduced lipid peroxidation and cellular damage. Similar antioxidant‐ and immune‐related improvements have been reported in other species, including Nile tilapia (*Oreochromis niloticus*), in which dietary SCZ powder increased lysozyme activity and reduced MDA levels [50] and largemouth bass (*Micropterus salmoides*), in which SCZ supplementation enhanced SOD activity [51]. Furthermore, in *S. salar*, replacement of FO with SCZ powder has been shown to promote astaxanthin accumulation in muscle tissue and upregulate the expression of antioxidant‐related genes, NK‐lysin, and component C3, thereby enhancing oxidative‐stress resistance [52]. The simultaneous enhancement of immune responses and antioxidant defenses suggests that dietary SCZO combined with CNO helps maintain the immune–redox balance under thermal stress conditions. Maintaining redox balance is critical as excessive ROS accumulation can trigger inflammatory cascades and subsequent cellular damage [53]. In this context, the downregulation of proinflammatory cytokines, including *TNF-α*, *IL-1β*, *IL-6*, and *MULAN*, observed in the present study indicates that dietary lipid replacement mitigated stress‐induced inflammatory responses [38]. These cytokines are key mediators of acute inflammation, and their overexpression under thermal stress has been closely associated with tissue damage and impaired physiological function [54]. In addition, DHA serves as a precursor for specialized proresolving mediators that actively regulate inflammatory responses and promote recovery from stress‐induced damage [55]. Oxidative stress and inflammation are also closely linked to apoptotic pathway activation [56]. The significant downregulation of apoptosis‐related genes, including *BAX*, *Caspase-3*, *Caspase-7*, *Caspase-9*, and *P53*, indicates that dietary SCZO combined with CNO reduced stress‐induced cell death. The mitochondrial apoptotic pathway is particularly sensitive to oxidative damage as elevated ROS levels can induce mitochondrial dysfunction and activate proapoptotic factors such as *BAX*, thereby initiating the caspase cascade [57]. Consequently, the reduced expression of apoptotic genes indicates that enhanced antioxidant capacity helped preserve cellular integrity by limiting ROS‐mediated mitochondrial damage. Furthermore, the downregulation of stress‐related genes such as HSPs suggests that fish in the replacement groups experienced reduced physiological stress [58]. Collectively, these findings indicate that replacing FO with SCZO combined with CNO enhances antioxidant and immune defenses as well as suppresses excessive inflammation and apoptosis under high‐temperature stress.

In the present study, dietary replacement of FO with SCZO and CNO markedly modulated lipid metabolism at the molecular and biochemical levels. The significant downregulation of lipogenic genes, including *FAS*, Δ*5D*, Δ*6D*, *LXR*, *SREBP1*, and *SREBP2*, indicates reduced de novo lipid synthesis [59]. *LXR* is a key regulator upstream of lipid metabolism, activating SREBP‐mediated fatty acid and CHOL biosynthesis pathways [60]. Therefore, the observed decrease in *LXR* expression likely contributed to the coordinated downregulation of these downstream lipogenic genes, ultimately suppressing hepatic lipid synthesis. Concurrently, the upregulation of genes involved in fatty acid catabolism, such as *ACOX1*, *CPT1*, *PPARα*, and *PPARβ*, suggests a metabolic shift toward enhanced lipid utilization [61]. This metabolic change was supported by the significant increase in hepatic *β*‐oxidation activity in the S40–S100 groups, confirming that fatty acid catabolism was functionally elevated rather than transcriptionally regulated alone. These coordinated responses suggest that dietary modification with SCZO and CNO promotes efficient energy metabolism under thermal stress. *β*‐Oxidation is essential for adenosine triphosphate production in fish, particularly when energy demands for maintenance, osmoregulation, and cellular repair are higher [62]. Therefore, the observed increase in *β*‐oxidation activity reflects enhanced energy‐generating capacity, likely contributing to improved growth and survival. The fatty acid composition of muscle tissue reflected dietary fatty acid profiles, with increased DHA and reduced EPA levels in fish fed SCZO‐containing diets. Enhanced DHA deposition is particularly important because DHA is preferentially incorporated into cell membranes and plays a key role in maintaining membrane integrity and function under stress conditions [33]. When dietary DHA is sufficient, fish can reduce their reliance on endogenous elongation and desaturation pathways, thereby lowering metabolic costs and conserving energy [63]. Moreover, the increase in crude protein content in the muscles of fish fed FO‐replaced diets suggests improved protein retention efficiency [64], which is potentially linked to enhanced lipid utilization as efficient *β*‐oxidation spares amino acids from use as an energy source, allowing their allocation for growth and tissue development [62]. Such a protein‐sparing effect may be particularly advantageous under high‐temperature stress, where metabolic inefficiency and maintenance energy demands are generally elevated. Collectively, the present findings indicate that replacing FO with the combination of SCZO and CNO induces a coordinated metabolic shift characterized by reduced lipogenesis, enhanced fatty acid *β*‐oxidation, and an improved fatty acid profile. These adaptations enhance energy utilization efficiency and help maintain metabolic homeostasis under thermal stress, thereby supporting improved growth performance and physiological resilience in *S. salar*.

High‐temperature stress disrupts intestinal microbial homeostasis in fish, reducing microbial diversity and promoting opportunistic pathogens, thereby impairing intestinal health, immune function, and nutrient absorption [65]. In the present study, replacing FO with SCZO and CNO markedly altered the intestinal microbial community, as evidenced by distinct clustering in PCoA and higher alpha diversity indices, including Chao1, Shannon, and Faith’s PD. Increased microbial diversity is considered a hallmark of a stable, resilient intestinal ecosystem, particularly under environmental stress [66]. The present results suggest that dietary SCZO combined with CNO helps maintain intestinal microbial stability under high‐temperature stress. Notably, Vibrionaceae and *Vibrio* abundances decreased in the FO replacement groups. Vibrionaceae, including *Vibrio* spp., are opportunistic pathogens, frequently associated with intestinal dysbiosis, inflammation, and stress‐related mortality in marine fish under high‐temperature conditions [67]. The markedly higher abundance of *Vibrio* observed in the S0 group may indicate a greater susceptibility to thermal stress–induced microbial imbalance. In contrast, the increased abundance of beneficial taxa, including *Lactobacillus*, *Bacillus*, and *Enterococcus*, in fish fed SCZO‐containing diets suggests an improvement in intestinal microbial composition. These genera contribute to intestinal health through the competitive exclusion of pathogens, production of antimicrobial compounds, and modulation of host immune responses [68]. In particular, *Lactobacillus* and *Bacillus* enhance intestinal barrier integrity and antioxidant defenses, improving stress resistance in fish under adverse environmental conditions [69]. These findings are consistent with those of previous studies reporting that SCZ supplementation increases the abundance of beneficial bacteria, such as *Lactobacillus*, while reducing potentially pathogenic taxa, including Vibrionaceae, thereby improving the intestinal microbial balance and host immune status [70]. The increased Lachnospiraceae abundance in FO‐replaced groups may also have physiological implications, as these bacteria produce short‐chain fatty acids (SCFAs), particularly butyrate, which support epithelial energy metabolism, intestinal barrier integrity, and antiinflammatory responses [71]. Their increase may partly explain the improved intestinal morphology, reduced vacuolization, and suppressed inflammatory responses observed in the present study. Overall, these findings highlight the interaction between dietary lipid composition, intestinal microbial modulation, and host physiological stability. Similarly, Shi et al. [17] reported that SCZ supplementation enriched SCFAs‐producing bacteria, including *Butyricicoccus* and *Dorea*, which were closely associated with intestinal homeostasis, immune regulation, and enteric pathogen resistance. The beneficial microbial shifts observed in the present study may be attributable, at least in part, to the high DHA content of SCZO. Dietary DHA has been reported to influence intestinal microbial composition by altering intestinal lipid environments, membrane characteristics, and inflammatory status, thereby promoting beneficial bacteria and suppressing opportunistic pathogens [72]. Additionally, improved intestinal integrity and reduced oxidative stress in SCZO‐fed fish may create a stable environment, supporting microbial diversity and community stability. Therefore, replacing FO with SCZO and CNO modulates intestinal microbial composition and promotes a healthier, more resilient intestinal ecosystem under high‐temperature stress, potentially improving growth, immune competence, and survival in *S. salar*.

## 5. Conclusion

The present study demonstrated that FO can be completely replaced by SCZO combined with CNO in diets for *S. salar* under high‐temperature stress without compromising the growth performance or physiological health. Replacement of FO with the combination of SCZO and CNO improved growth and feed efficiency, while survival increased progressively with increasing FO replacement. These benefits were associated with enhanced digestive capacity, improved intestinal morphology, stabilized hematological parameters, and strengthened immune and antioxidant defenses. In addition, dietary replacement induced a coordinated metabolic shift characterized by reduced lipogenesis, enhanced fatty acid *β*‐oxidation, and increased DHA deposition in muscle tissue, indicating improved energy utilization efficiency and metabolic homeostasis under thermal stress. Furthermore, SCZO‐containing diets modulated intestinal microbial composition by increasing beneficial bacterial taxa and reducing opportunistic pathogens such as *Vibrio*. Collectively, these findings suggest that the high DHA supply associated with increasing SCZO inclusion may have contributed to maintaining immune–redox balance, intestinal integrity, and metabolic resilience during high‐temperature stress, supporting the potential of SCZO combined with CNO as an alternative to FO in *S. salar* diets.

## Author Contributions

**Suhyeok Kim**: writing – original draft, formal analysis, data curation. **Hyunwoon Lim** and **Jin-Young Jeon:** resources and conceptualization. **Kyeong-Jun Lee**: writing – review and editing, supervision, methodology, conceptualization.

## Funding

This research was supported by the Korea Institute of Marine Science & Technology Promotion (KIMST) funded by the Ministry of Oceans and Fisheries (Grant RS‐2025‐04262969) and the National Research Foundation of Korea (NRF) grant funded by the Korea government (MSIT) (Grant RS‐2026‐25488677).

## Disclosure

The company (Daesang Corporation) had no role in the study design, data collection, data analysis, interpretation of the results, or preparation of the manuscript.

## Ethics Statement

All experimental procedures involving Atlantic salmon (*Salmo salar*) were approved by the Institutional Animal Care and Use Committee (IACUC) of Jeju National University (Approval Number 2025‐0068) and conducted in accordance with relevant guidelines and regulations.

## Conflicts of Interest

The authors declare no conflicts of interest.

## Supporting Information

Additional supporting information can be found online in the Supporting Information section.

## Supporting information


**Supporting Information** Table S1: Oxidative quality parameters of the experimental oil sources. Figure S1: Representative histological images of intestinal morphology in Atlantic salmon *Salmo salar* used for morphometric analysis. (A) Cross‐sectional overview of the intestine. (B) Measurement of villi width (a) and villi length (b). (C) Representative vacuolization within enterocytes (c). Scale bars = 500 *μ*m in (A), 200 *μ*m in (B), and 50 *μ*m in (C).

## Data Availability

All datasets analyzed in this study are available from the corresponding author upon reasonable request.
