# Supplementary material for: Combined Schizochytrium and Canola Oil Effectively Replaces Fish Oil in Atlantic Salmon (Salmo salar) Under Chronic Thermal Stress
Source: Aquac Nutr. 2026 Oct 30;2026:8779572. doi: 10.1155/anu/8779572 (PMC13720513; doi:10.1155/anu/8779572)
Supplement: Supplementary file 1 [file ANU-2026-8779572-s001.docx]

**Table S1.** Oxidative quality parameters of the experimental oil sources

| Lipid sources | PV (meq O₂/kg oil)^1^ | p-AV^2^ | TOTOX^3^ |
| --- | --- | --- | --- |
| FO^4^ | 3.88±0.05 | 4.35±0.26 | 12.12±0.19 |
| CNO^5^ | 3.93±0.08 | 4.72±0.21 | 12.57±0.05 |
| SCZO^6^ | 3.21±0.06 | 4.11±0.12 | 10.54±0.08 |

Values are presented as mean ± S.D. (n = 3). ^1^PV, peroxide value; ^2^p-AV, p-anisidine value; ^3^TOTOX, total oxidation value. TOTOX was calculated as 2 × PV + p-AV. The maximum limits for fish oils according to the Codex Standard for Fish Oils (CXS 329-2017) are PV ≤ 5 meq active oxygen/kg oil, p-AV ≤ 20 and TOTOX ≤ 26. ^4^Fish oil (cod liver oil); ^5^Canola oil; ^6^*Schizochytrium* oil.

**Fig. S1.** Representative histological images of intestinal morphology in Atlantic salmon *Salmo salar* used for morphometric analysis. (A) Cross-sectional overview of the intestine. (B) Measurement of villi width (a) and villi length (b). (C) Representative vacuolization within enterocytes (c). Scale bars = 500 μm in (A), 200 μm in (B), and 50 μm in (C).
